# Does Pelvic Fixation in Neuromuscular Scoliosis Correction Affect Radiological and Functional Outcomes?

**DOI:** 10.7759/cureus.88148

**Published:** 2025-07-17

**Authors:** Rodrigo Muscogliati, Rawan Masarwa, Elie Najjar, Mohammad Daher, Mostafa Meshneb, Ankur Goswami, Ahmed A Hassan, Nasir A Quraishi, Mohammed S Patel

**Affiliations:** 1 Hull York Medical School, University of Hull, Hull, GBR; 2 Centre for Spinal Studies and Surgery, Queens Medical Centre, Nottingham University Hospitals National Health Service (NHS) Trust, Nottingham, GBR

**Keywords:** functional outcomes, neuromuscular spine, pelvic fixation, pelvic obliquity, radiological outcomes

## Abstract

Pelvic fixation is commonly used in neuromuscular scoliosis patients with pelvic obliquity (PO) >15°, yet its benefit over shorter constructs remains unclear. This systematic review analyzed five studies comprising 230 patients (120 with pelvic fixation (WPF), 110 without pelvic fixation (WoPF)) to assess radiological, functional, and surgical outcomes. Radiographic correction was similar between groups: Cobb angle improved from 62.8° to 32.4° in WPF vs. 76.7° to 29.4° in WoPF (p = 0.54), and PO correction did not differ significantly (final PO 8.6° vs. 11.5°, p = 0.5). Functional outcomes were heterogeneous; only one study reported a statistically significant benefit in the WPF group (Short-Form Health Survey-36 improvement, p = 0.007), while others using Gross Motor Function Classification System (GMFCS), Caregiver Priorities and Child Health Index of Life With Disabilities, or Bridwell’s scores found no advantage. GMFCS data were reported in just 19% of patients, limiting subgroup analysis. Estimated blood loss was substantially higher in WPF (2,851 mL vs. 1,383 mL), though not statistically significant (p = 0.17), with comparable complication rates. Given the heterogeneity in outcome measures, surgical technique, and patient function, routine pelvic fixation cannot be universally recommended. However, it may benefit select subgroups, such as non-ambulatory patients with severe PO or poor trunk control, warranting individualized surgical planning.

## Introduction and background

Neuromuscular scoliosis (NMS) is a complex spinal deformity resulting from an underlying neuromuscular disorder that impairs motor control and posture. Common causes include cerebral palsy (CP), Duchenne muscular dystrophy (DMD), spinal muscular atrophy (SMA), and other syndromic or acquired neuromuscular conditions. The severity of NMS correlates with the extent of neurological impairment and is often greater in non-ambulatory patients [1,2].

Pelvic obliquity (PO), a frequent and progressive complication of NMS, arises from asymmetrical muscle forces, hip contractures, and uneven spinal loading. This leads to spinal curvature progression, sitting imbalance, increased risk of pressure ulcers, and compromised respiratory function, collectively reducing quality of life [3-5].

Surgical correction is generally indicated for curves exceeding 40°-50°, aiming to achieve a stable, well-aligned spine over a level pelvis [6]. Pelvic fixation, often using iliac screws, is typically recommended when PO exceeds 15°, as it is thought to enhance coronal and sagittal alignment and maintain long-term correction [7,8]. However, pelvic fixation is associated with higher surgical morbidity, including greater blood loss, longer operative times, and increased risk of complications such as wound issues and hardware failure [9,10]. Additionally, in partially ambulatory patients, fusing to the pelvis may reduce residual trunk mobility, potentially impacting function [11,12].

Biomechanically, the pelvis serves as a critical transition point between the spine and lower extremities [13]. Surgical decisions must consider ambulatory status, pelvic balance, and fixed contractures. Some studies suggest that fusion short of the pelvis may suffice in select cases with minimal PO and preserved mobility [14]. However, the lack of standardized criteria for pelvic fixation and inconsistent outcome measures across studies limits the generalizability of findings [9].

The objective of this systematic review is to evaluate whether pelvic fixation in NMS patients with PO >15° improves clinical and radiological outcomes, including Cobb angle correction, pelvic tilt reduction, complication rates, blood loss, hospital stay, and functional status. Commonly used instruments to assess functional outcomes include the Caregiver Priorities and Child Health Index of Life With Disabilities (CPCHILD), Short Form Health Survey-36 (SF-36), Gross Motor Function Classification System (GMFCS), and Bridwell’s questionnaire. Therefore, the research question of this review is: “Does pelvic fixation improve radiological or functional outcomes in NMS patients compared to fusion short of the pelvis?” This evidence synthesis aims to inform surgical decision-making and identify which subgroups may derive the most benefit from pelvic fixation.

## Review

Methodology

Search Strategy

This systematic review adhered to the Preferred Reporting Items for Systematic Reviews and Meta-Analyses (PRISMA) guidelines [15]. A comprehensive literature search was performed using PubMed, Medline, and Embase databases, covering studies available through August 2024. The search strategy was not limited by language, publication date, or publication type. Although non-English-language studies were not excluded during the search phase, none met the inclusion criteria after full-text review.

The following Boolean search strategy was used (maintained in its original form to maximize sensitivity): ((Cerebral palsy) OR (Brain palsy) OR (Central palsy) OR (Cerebral paresis) OR (Diplegia spastica) OR (Encephalopethia infantilis) OR (Neuromuscular) OR (“Cerebral Palsy”[Mesh])) AND ((Scoliosis) OR (“Scoliosis”[Mesh])) AND ((Pelvis) OR (Pelvic) OR (“Pelvis”[Mesh])) AND ((Fusion) OR (Fixation) OR (Stabilization) OR (Surgical Management) OR (Surgical Correction) OR (“Spinal Fusion”[Mesh])).

CP was included as a specific term because it represents the most common cause of NMS and is frequently used as a clinical model in surgical outcome studies, thereby justifying its inclusion for generalizability to other neuromuscular conditions.

Duplicate articles were removed before screening. Two researchers independently screened titles and abstracts. Full-text screening was then conducted for studies that passed the initial screening using the same inclusion and exclusion criteria. Disagreements between reviewers were resolved by a third researcher.

Inclusion and Exclusion Criteria

The following studies were considered for inclusion in the review: studies focusing on NMS patients, studies in which surgical scoliosis correction was performed with or without fixation to the pelvis, and studies reporting outcomes related to the pre- and postoperative function. We excluded case reports, conference abstracts, studies in which outcomes were not specific to surgeries with pelvic fixation, and studies in which outcomes regarding ambulation were not reported.

Non-English Studies and Grey Literature

Non-English studies were excluded during abstract screening due to limitations in translation resources, which could affect the accuracy and consistency of data extraction and analysis. Grey literature, including conference abstracts and unpublished data, was excluded to maintain methodological rigor, ensure quality control through peer review, and enhance the reproducibility of findings. While this may limit the inclusion of some potentially relevant data, it aligns with established standards for systematic reviews aiming to synthesize clinically actionable evidence.

Data Extraction

Data extraction was conducted independently by two researchers. For each included study, the following variables were collected: first author, publication year, level of evidence, number of patients, patient demographics, average blood loss, hospital stay duration, pre- and postoperative Cobb angle and pelvic tilt, major complications, and pre- and postoperative functional and ambulatory outcomes.

Statistical Analysis

Statistical analyses were performed using SPSS software (version 26.0; IBM Corp., Armonk, NY, USA). Continuous variables were assessed using the Student’s t-test, and categorical variables with the chi-square test. Binary logistic regression was used to evaluate the independent effect of pelvic fixation on postoperative radiological outcomes. Heterogeneity across studies was assessed using the I² statistic, with several key outcomes having values >50% indicating moderate to high heterogeneity. Additional variation in follow-up durations, surgical technique (e.g., use of sacral alar-iliac (SAI) screws vs. traditional iliac fixation), and baseline curve severity further limited the comparability of radiological outcomes. Functional outcomes were assessed using four different instruments across studies (GMFCS, CPCHILD, SF-36, and Bridwell’s questionnaire). Given these methodological and clinical inconsistencies, a formal meta-analysis was deemed inappropriate. Instead, a narrative synthesis was conducted to compare findings across studies. A p-value <0.05 was considered statistically significant.

Results

Study Selection and Evidence Level

**Figure 1:**
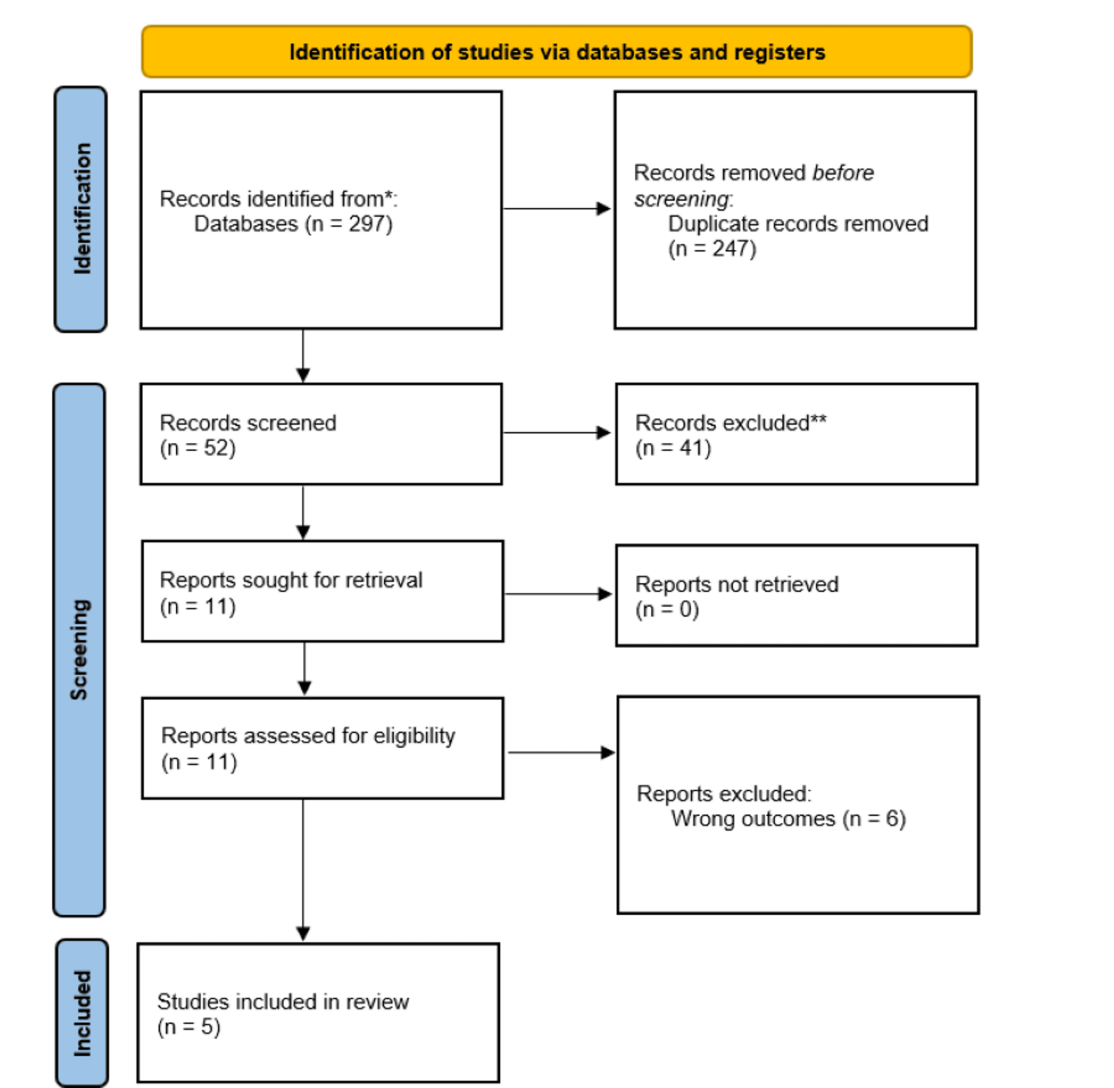
Preferred Reporting Items for Systematic Reviews and Meta-Analyses (PRISMA) flowchart depicting the screening process to identify the five papers included in this review.

Out of 297 articles initially screened, five studies met the inclusion criteria and were ultimately included in the analysis, as depicted in the PRISMA flowchart in Figure 1 [15]. According to the Oxford Centre for Evidence-Based Medicine guidelines, all five studies were classified as Level III evidence [16].

Risk of Bias Assessment

Risk of bias assessment was performed using the Joana Briggs Institute (JBI) critical appraisal checklist questionnaire [17]. Four cohort studies, Farshad et al. (2022) [12], Badin et al. (2023) [11], Yang et al. (2023) [8], and Akesen et al. (2018) [18], were appraised using the JBI Checklist for Cohort Studies. All studies clearly defined exposures and used valid, reliable outcome measures with adequate follow-up periods, as depicted in Table 1. The PICO of the five included studies is presented in Table 2.

**Table 1:** Joana Briggs Institute quality assessment of cohort studies. Q1: Were the two groups similar and recruited from the same population? Q2: Were the exposures measured similarly to assign people to groups? Q3: Was the exposure measured in a valid and reliable way? Q4: Were confounding factors identified? Q5: Were strategies to deal with confounding factors stated? Q6: Were participants free of the outcome at the start? Q7: Were outcomes measured in a valid and reliable way? Q8: Was the follow-up time sufficient for outcomes to occur? Q9: Was the follow-up complete, and reasons for loss described? Q10: Were the strategies to address incomplete follow-up used? Q11: Was appropriate statistical analysis used?

Study	Q1	Q2	Q3	Q4	Q5	Q6	Q7	Q8	Q9	Q10	Q11	Overall appraisal
Farshad et al. (2022) [12]	Yes	Yes	Yes	Yes	Yes	Yes	Yes	Yes	Yes	N/A	Yes	Include
Badin et al. (2023) [11]	Yes	Yes	Yes	Yes	Yes	Yes	Yes	Yes	Yes	Yes	Yes	Include
Yang et al. (2023) [8]	Yes	Yes	Yes	Yes	Yes	Yes	Yes	Yes	Yes	N/A	Yes	Include
Akesen et al. (2018) [18]	Yes	Yes	Yes	Unclear	No	Yes	Yes	Yes	Yes	N/A	Yes	Include

**Table 2:** Characteristics of the included studies. PO = pelvic obliquity; CPCHILD = Caregiver Priorities and Child Health Index of Life With Disabilities; SF-36 = Short Form Health Survey-36; GMFCS = Gross Motor Function Classification System

Study	Population	Intervention	Comparison	Outcome
Baymurat et al. (2023) [19]	33 patients aged 10–20 years with PO >15°, ≥2 years follow-up	Posterior spinal fusion with pelvic fixation	Without pelvic fixation	No significant differences in GMFCS scores, radiological correction, or activity levels
Farshad et al. (2022) [12]	49 wheelchair-bound patients (GMFCS IV–V), mean Cobb angle 70°, PO 20°	Fusion including pelvic fixation (alar-iliac screws)	Without pelvic fixation	No significant differences in complications, GMFCS improvement (mean +0.5), or revision rates
Badin et al. (2023) [11]	92 children with cerebral palsy and sitting balance, PO <17°	Fusion to the pelvis	Fusion short of the pelvis	No difference in CPCHILD scores at 2 and 5 years; shorter fusion safe in those with trunk control
Yang et al. (2023) [8]	77 patients (mostly GMFCS IV–V); grouped by fixation level (pelvis, S1, L5)	Spino-pelvic fixation with iliac screws	Fixation to S1 or L5	No significant differences in scoliosis/PO correction or functional outcomes
Akesen et al. (2018) [18]	38 non-ambulatory patients undergoing posterior spinal fusion	Fusion with iliac screw fixation	Without iliac screws	Significant SF-36 physical function improvement in fixation group (p = 0.007)

The studies by Farshad et al., Badin et al., and Yang et al. met all key criteria, including identification and control of confounding factors through appropriate statistical analyses and complete follow-up. Akesen et al. met most criteria but did not clearly report strategies for dealing with confounders. Despite this, it used valid outcome measures and had adequate follow-up. All were judged suitable for inclusion.

The study by Baymurat et al. (2023) [19] was assessed using the JBI Case-Control Checklist, as shown in Table 3. The study featured well-matched groups, consistent and valid exposure/outcome measurement, and clear confounder control via multivariate analysis. Follow-up was sufficient, and statistical analysis was appropriate. It was deemed high quality and suitable for inclusion.

**Table 3:** Joana Briggs Institute quality assessment of the case-control study. Analysis of the study by Baymurat et al. [19].

Checklist Item	Response
1. Were the groups comparable other than the presence of disease in cases or absence in controls?	Yes
2. Were the cases and controls matched appropriately?	Yes
3. Were the same criteria used for the identification of cases and controls?	Yes
4. Was the exposure measured in a standard, valid, and reliable way?	Yes
5. Was the exposure measured in the same way for cases and controls?	Yes
6. Were the confounding factors identified?	Yes
7. Were the strategies to deal with confounding factors stated?	Yes
8. Were the outcomes assessed in a standard, valid, and reliable way for cases and controls?	Yes
9. Was the exposure period of interest long enough to be meaningful?	Yes
10. Was appropriate statistical analysis used?	Yes
Overall appraisal	Include

Demographics and Study Characteristics

The included studies encompassed a total of 230 patients, with 120 patients undergoing surgery with pelvic fixation (WPF) and 110 patients receiving spinal fusion without pelvic fixation (WoPF). The average age of patients in the WPF group was 15.8 years (range = 10-24 years, male-to-female ratio = 0.87), while in the WoPF group, it was 15.6 years (range = 9-25 years, male-to-female ratio = 1.08). The average duration of follow-up was 30 months (range = 24-37 months) in the WPF group and 36 months (range = 25-50 months) in the WoPF group.

Among the included studies that reported GMFCS levels [12,19], data were available for 44 patients, accounting for 19% of the total population. The distribution was as follows: GMFCS Level I, 11.4% (5/44); Level II, 11.4% (4/44); Level III, 13.6% (6/44); Level IV, 40.9% (18/44); and Level V, 22.7% (10/44).

Surgical Outcomes

Regarding intraoperative and immediate postoperative metrics, the average estimated blood loss was higher in the WPF group at 2,851 mL, compared to 1,383 mL in the WoPF group. Despite this difference, it did not reach statistical significance (p = 0.17). Hospital stay duration was also similar between groups, averaging 19 days in the WPF cohort and 21 days in the WoPF cohort (p = 0.67). Major postoperative complications were reported in 29 patients within the WPF group and 34 patients in the WoPF group, again without a statistically significant difference (p = 0.55).

Functional Outcomes

Functional outcomes were assessed using various validated instruments across the included studies, such as CPCHILD, SF-36, GMFCS, and Bridwell’s questionnaire. However, variability in outcome measures and baseline patient characteristics limited direct comparisons. Most studies found no significant functional benefit attributable specifically to pelvic fixation, with only the study by Akesen et al. [18] reporting a statistically significant improvement in physical functioning using the SF-36. Collectively, these findings suggest that pelvic fixation does not consistently improve functional status across the broader NMS population.

Baymurat et al. [19] evaluated function using GMFCS levels and found no significant postoperative changes in either group. The WPF group had a mean GMFCS level of 3.76 compared to 2.75 in the WoPF group, suggesting no clear benefit of pelvic fixation on gross motor function. Similarly, Farshad et al. [12] reported a mean improvement of 0.5 GMFCS points across all patients; however, there was no statistically significant difference between the WPF and WoPF groups.

Badin et al. [11] assessed health-related quality of life using the CPCHILD questionnaire. At two years postoperatively, personal care and mobility domain scores were 69.3 ± 12.3 in the WPF group and 60.3 ± 13.9 in the WoPF group (p = 0.122). At five years, scores were 61.2 ± 17.9 and 63.9 ± 11.6, respectively (p = 0.766), indicating no long-term functional advantage from pelvic fixation.

Yang et al. [8] used Bridwell’s questionnaire to assess postoperative function and patient satisfaction. Their results showed no significant differences between groups. All patients in this study were non-ambulatory, limiting the scope of functional outcomes to seated balance and quality of life metrics.

Only Akesen et al. [18] reported a statistically significant difference favoring pelvic fixation. Their study, which utilized the SF-36, found that the WPF group experienced greater improvement in the physical component score (7.6 vs. 3.6, p = 0.007). This suggests that in selected cases, pelvic fixation may confer measurable physical function benefits.

To provide a consolidated overview of the functional findings from the included studies, a summary is presented in Table 4.

**Table 4:** Pre- and postoperative function in WPF and WoPF groups. CPCHILD = Caregiver Priorities and Child Health Index of Life With Disabilities; SF-36 = Short Form Health Survey; GMFCS = Gross Motor Function Classification System; WPF = with pelvic fixation; WoPF = without pelvic fixation

Study	Index of function used	Preoperative function	Postoperative function (WoPF)	Postoperative function (WPF)
Badin et al. (2023) [11]	CPCHILD	Not reported	2 years = 60.3 ± 13.9; 5 years = 63.9 ± 11.6	2 years = 69.3 ± 12.3; 5 years = 61.2 ± 17.9
Akesen et al. (2018) [18]	SF-36	WoPF = 54 (33–68); WPF = 55 (34–61)	54 (36–70)	60 (39–88)
Baymurat et al. (2023) [19]	GMFCS	WoPF = 2.75 ± 1.29; WPF = 3.76 ± 1.03	2.75 ± 1.29	3.76 ± 1.03
Farshad et al. (2022) [12]	GMFCS	WoPF = 4.72; WPF = 4.67	0.8 ± 0.4 improvement	0.6 ± 0.5 improvement
Yang et al. (2023) [8]	Bridwell’s Questionnaire	Not reported	Fixed to L5 = 0.0 ± 0.6; fixed to S1 = 0.1 ± 0.6	0.1 ± 0.6

Ambulatory Status

Ambulatory status data, though sparsely reported, are summarized in Table 5. These indicate no changes in pre- versus postoperative status across the studies that reported this outcome. Most notably, all patients in the study by Akesen et al. [18] were non-ambulatory, and no shift was noted postoperatively. Badin et al. [11] also observed no change in seated independence across their cohort.

**Table 5:** Ambulatory status pre- and postoperatively in WPF and WoPF groups. WPF = with pelvic fixation; WoPF = without pelvic fixation

Study	Preoperative ambulation	Postoperative ambulation
Badin et al. (2023) [11]	38 with independent sitting; 48 without	38 with independent sitting; 48 without
Akesen et al. (2018) [18]	All patients non-ambulatory	All patients non-ambulatory
Baymurat et al. (2023) [19]	WoPF = 7 non-ambulatory; WPF = 10 non-ambulatory	Same as the preoperative status
Farshad et al. (2022) [12]	WoPF = 11 non-ambulatory; WPF = 9 non-ambulatory	Not reported
Yang et al. (2023) [8]	Not reported	Not reported

These findings reinforce that functional and ambulatory outcomes remain largely unchanged by the addition of pelvic fixation in NMS surgery, suggesting that other factors, such as baseline function and sitting ability, may play more decisive roles in outcome prediction.

Radiological Outcomes

Radiographic measures also failed to show statistically significant differences between groups. The mean preoperative Cobb angle in the WPF group was 62.8°, which improved to 32.4° at the two-year follow-up. In the WoPF group, the angle improved from 76.7° preoperatively to 29.4° at follow-up. There was no significant difference between groups in either preoperative (p = 0.06) or two-year Cobb angle (p = 0.54).

PO showed similar trends. Preoperative PO was 16.5° in the WPF group and 16.8° in the WoPF group (p = 0.3). At the two-year follow-up, values improved to 8.6° in the WPF group and 11.5° in the WoPF group, with no statistically significant difference between groups (p = 0.5).

Discussion

Pelvic fixation in NMS remains a nuanced component of surgical planning. While theoretical benefits include improved PO correction, seated balance, and long-term spinal alignment, our systematic review did not find consistent evidence supporting universal use. However, this lack of consistency should not be interpreted as evidence of no benefit; rather, the value of pelvic fixation may be limited to select patient subgroups [8,12].

Functional Outcomes

Among the five included studies, most found no statistically significant difference in radiological or functional outcomes between pelvic fixation (WPF) and non-pelvic fixation (WoPF) cohorts. For instance, Baymurat et al. [19], Farshad et al. [12], and Yang et al. [8] observed no meaningful GMFCS or radiographic advantages with pelvic fixation. Badin et al. [11] similarly found comparable CPCHILD scores at two and five years, suggesting that baseline function, not fixation level, may determine postoperative trajectory.

However, Akesen et al. [18] reported a significant improvement in SF-36 physical scores in the WPF group, particularly among non-ambulatory patients. This discrepancy may reflect differences in patient selection (e.g., GMFCS IV-V), assessment tools, or surgical technique. It highlights the need to avoid overly broad generalizations and instead consider the possibility of subgroup-specific benefits, particularly for patients with poor trunk control or severe PO.

The heterogeneity in functional outcomes was likely driven by variability in baseline ambulatory status, use of different measurement tools (GMFCS vs. CPCHILD vs. SF-36), and inconsistent follow-up duration. Although these limitations precluded formal subgroup analysis, emerging patterns suggest that non-ambulatory patients with severe PO may benefit more consistently from pelvic fixation.

Radiological Outcomes

Radiologically, Cobb angle and PO correction were not significantly different between WPF and WoPF groups in most studies, corroborating previous reports such as Whitaker et al. [14] and Yang et al. [8]. Although Sponseller et al. [10] found improved PO correction using the SAI technique, this did not uniformly translate into functional gains. These findings support the selective use of pelvic fixation, particularly in patients with PO >15°, fixed contractures, or GMFCS levels IV-V, while fusion short of the pelvis may be appropriate for ambulant individuals with minimal obliquity.

Surgical Outcomes and Clinical Considerations

Of note, although the difference in estimated blood loss between groups did not reach statistical significance, the nearly two-fold increase observed in the pelvic fixation group (2,851 mL vs. 1,383 mL) is clinically relevant. In NMS populations, particularly those with poor nutritional status or cardiorespiratory disease, higher intraoperative blood loss can increase the risk of requiring blood transfusions, infection, and prolonged hospitalization. Surgeons should consider this when weighing the risks and benefits of extending constructs to the pelvis, especially in patients with borderline health reserves or limited physiological resilience.

It is important to contextualize these findings within the broader scope of NMS etiologies. As CP is the most common cause of NMS, several included studies focused exclusively on CP patients. However, the natural history and postoperative goals differ between CP and other conditions such as DMD or SMA. Functional expectations, curve progression, and trunk control vary by diagnosis. Therefore, while CP data is broadly representative, interpretation across the NMS spectrum should be approached with diagnostic nuance.

The technical aspects of fixation also influence outcomes. The use of SAI screws versus traditional iliac bolts affects complication profiles, such as soft tissue prominence, pseudoarthrosis, and surgical time. As noted by Sponseller et al. [10], SAI fixation may offer biomechanical and cosmetic advantages, though it requires specific expertise.

Collectively, the findings do not support routine pelvic fixation in all NMS cases. Instead, a selective, criteria-based approach is warranted, factoring in PO severity, GMFCS level, residual trunk control, and ambulatory status. This mirrors the perspective of Whitaker et al. [14], who cautioned against pelvic fixation in high-functioning individuals with minimal deformity.

Future Research

Given the variability in fixation strategies, outcome metrics, and patient subgroups, future studies should adopt standardized functional and radiological tools (e.g., CPCHILD, SF-36, GMFCS). Prospective, multi-center trials stratified by diagnosis and functional level would help clarify indications for pelvic fixation. Long-term follow-up and complication tracking are essential to guide evidence-based surgical decision-making.

Limitations

This review has several limitations. First, study heterogeneity was notable, particularly in terms of diagnosis (e.g., CP vs. DMD), functional scores used, and surgical techniques. This variability complicates pooled analysis and generalizability.

Functional outcomes were assessed with multiple instruments, i.e., GMFCS, CPCHILD, SF-36, and Bridwell’s, introducing interpretive inconsistency. Similarly, radiological endpoints such as PO correction were variably defined and measured. These differences precluded a formal meta-analysis. Furthermore, GMFCS levels were reported in only 44 of the 230 patients (19%) across the included studies. This limited availability of functional classification weakens the conclusions that can be drawn regarding functional outcomes, particularly as GMFCS level is a critical stratification variable in neuromuscular populations. The lack of comprehensive functional data also restricted our ability to conduct meaningful subgroup analyses.

Additionally, functional outcomes could not be standardized or pooled due to inconsistent reporting across studies. The included studies utilized different tools, GMFCS, SF-36, CPCHILD, and Bridwell’s questionnaire, with most reporting only categorical or ordinal data. Only one study provided a quantifiable SF-36 score [18]. As a result, conversion to standardized mean differences or percentage-based comparisons was not feasible, and a narrative synthesis was used instead.

Furthermore, few studies provided detailed long-term follow-up, limiting conclusions about the durability of surgical outcomes. While we attempted to synthesize results across studies, diagnostic heterogeneity remains an important confounder. As outcomes may differ substantially between NMS subtypes, caution is advised when applying findings broadly across this diverse population.

Lastly, all five included studies were classified as Level III evidence [16], representing retrospective cohort designs without randomization. This is a critical limitation, as systematic reviews based on lower-level evidence inherently carry a higher risk of bias and reduced internal validity. Without prospective designs or randomized control groups, the ability to draw causal inferences is limited. Consequently, while this review synthesizes available data, its conclusions should be interpreted with caution.

## Conclusions

This systematic review found no consistent evidence that pelvic fixation universally improves radiological or functional outcomes compared to shorter constructs in NMS surgery. While measures such as Cobb angle correction, PO, complication rates, and functional scores did not consistently favor pelvic fixation, one study did show a statistically significant improvement in physical function, specifically in patients with greater disability. These findings support a selective approach to pelvic fixation. In patients with severe PO (>15°), poor trunk control, or GMFCS levels IV-V, pelvic fixation may offer biomechanical or functional benefits. Conversely, in ambulatory individuals or those with minimal pelvic asymmetry, fusion short of the pelvis may achieve similar outcomes with fewer complications and less blood loss. Thus, surgical planning should be guided by objective clinical indicators, such as PO measurement, GMFCS classification, and seated balance, rather than routine inclusion of pelvic fixation. Given the retrospective nature and clinical heterogeneity of current evidence, future research should focus on prospective, multicenter studies stratified by underlying neuromuscular condition (e.g., CP, DMD) and functional level. Standardizing outcome tools (e.g., CPCHILD, SF-36, GMFCS) and including long-term follow-up will be essential for determining which patients truly benefit from pelvic fixation. Until then, decisions should prioritize individualized patient anatomy, function, and surgical goals.
